# Characterization of Background Exposures to Ethylene Oxide in the United States: A Reality Check on Theoretical Health Risks for Potentially Exposed Populations near Industrial Sources

**DOI:** 10.3390/ijerph22040597

**Published:** 2025-04-10

**Authors:** Christopher R. Kirman, Patrick J. Sheehan, Abby A. Li, James S. Bus, Steave H. Su, Pamela J. Dopart, Heather N. Watson, Emma E. Moynihan, Rick Reiss

**Affiliations:** 1 SciPinion, Bozeman, MT 59722, USA; 2 Health Sciences, Exponent, Inc., Oakland, CA 94612, USA; psheehan@exponent.com; 3 Health Sciences, Exponent, Inc., Alexandria, VA 22314, USA; abbyli@exponent.com (A.A.L.); jbus@exponent.com (J.S.B.); rreiss@exponent.com (R.R.); 4 Health Sciences, Exponent, Inc., New York, NY 10017, USA; ssu@exponent.com; 5 Health Sciences, Exponent, Inc., Bowie, MD 20715, USA; pdopart@exponent.com (P.J.D.); emoynihan@exponent.com (E.E.M.); 6 Data Science, Exponent Inc., Bellevue, WA 98007, USA; hwatson@exponent.com

**Keywords:** ethylene oxide, risk specific concentrations, total and endogenous equivalent background concentrations, managing and communicating risk

## Abstract

Ethylene oxide (EO) is an industrial chemical and sterilant that is released into ambient air from natural and unregulated anthropogenic sources that contribute to background exogenous exposure and from regulated industrial sources that contribute to additional exogenous exposure for near-facility populations. Metabolic processes contribute to substantial background endogenous exposures to EO, complicating the interpretation of the relation between total background exposure and the health significance of added industrial exogenous exposure. In 2021, Kirman and colleagues characterized the total and endogenous equivalent background concentrations for U.S. populations, which are substantially greater than the USEPA 2016 EO cancer reassessment risk-specific concentrations (0.00011–0.011 ppb), suggesting that the consideration of background exposure could be used as a reality check for the utility of the reassessment in managing EO risk for industrially exposed populations. New exposure biomarker data and background ambient concentration data for EO have become available since the 2021 assessment and are used here to refine the estimates of U.S. population total and endogenous equivalent background EO concentrations. Refined equivalent background concentrations as well as total equivalent exposure estimates for U.S. smokers provide context as to the health significance of near-industry population added exposure and a reality check for the utility of USEPA and TCEQ risk-specific concentrations in managing and communicating EO risk.

## 1. Introduction

Ethylene oxide (EO) is an industrial chemical that is used in the synthesis of other chemicals and, importantly, in the sterilization of medical equipment and supplies. Exposures to EO in the U.S. are widespread, originating from a variety of background sources that include natural plant and microbial releases of ethylene (ET), human endogenous processes, sources of combustion (e.g., cigarette smoke), and in addition, regulated releases associated with its industrial use [1]. As a result of the U.S. Environmental Protection Agency’s (USEPA’s) 2016 reassessment of the cancer potency of EO [2], estimates for the potential cancer risks resulting from EO exposures have increased by approximately 50-fold. This change in potency results in a reciprocal change in the exposures considered to pose an unacceptable risk (i.e., low-risk specific concentrations of 0.00011 ppb to 0.011 ppb for 10^−6^ to 10^−4^ risks) intended to support the management and communication of health risks for exposed populations. In contrast, a substantially lower cancer potency estimate for EO (and higher risk-specific concentrations; 0.24 to 24 ppb for 10^−6^ to 10^−4^ risks) was subsequently derived by the Texas Commission on Environmental Quality (TCEQ) [3], who relied on the same underlying epidemiology National Institute for Occupational Safety and Health (NIOSH) cohort and exposure data as USEPA.

The USEPA cancer potency estimate is based on breast and lymphoid cancers, with lymphoid cancer contributing approximately 87% to the total cancer potency estimate [2]. The TCEQ cancer potency estimate is based solely on lymphoid cancers because TCEQ [3] concluded that the full weight of the epidemiological evidence does not support the conclusion that EO causes breast cancer in humans. However, the primary reason for these differences is that USEPA and TCEQ utilized different statistical models to analyze the NIOSH data. This is evident by the approximately 260-fold difference between the unit risk estimate (URE) 2-piece linear spline model and standard log-linear Cox proportional hazards model, respectively, using USEPA IRIS methods [2] (The upper-bound unit risk (per ppm) for EPA’s model is 5.26 based on EPA IRIS (Tables 4–7). The upper-bound unit risk for the standard log-linear model is 0.020 based on EPA IRIS (2016)-derived equations and parameter estimates for the standard log-linear model using life table analysis for lymphoid incidence (EPA IRIS Table D-32) [2]).

Importantly, neither of the continuous models for lymphoid cancers are statistically significantly different from the null hypothesis [3]. Based on statistics alone, the standard log-linear Cox proportional hazards models fit the data just as well as the supralinear 2-piece spline slope for both lymphoid mortality and breast cancer incidence but have the advantage of parsimony (simpler model) and biological relevance [1]. USEPA [2] selected their model based on statistical and visual fit comparisons with four categorical (grouped) estimates, while TCEQ [3] emphasized biological plausibility, consistency with the conclusions of the original NIOSH publication by Steenland et al. [4], statistical fitting criteria to individual data that considered all statistical parameters modeled, and a reality check of the model prediction estimates.

Although these and other differences in statistical modeling methods continue to be debated in the public domain [3,5], less attention has been given to how new data on background EO exposures can help inform risk management decisions and provide a reality check for the risk assessment. The emphasis of this present investigation is to focus on how EO levels in the air, in smokers, and in the general population can inform risk management decisions for EO. The consideration of background exposures is a well-accepted risk management approach for the regulation of soil contaminates that are both naturally present and anthropogenically produced [6]. EPA [6] specifies that the difference from the background mean must be “sufficiently large to warrant additional interest based on health or ecological information” (i.e., an action level should be related to health or ecological risk in a meaningful way to inform mitigation decisions). Conceptually, this approach is directly relevant for the risk management of air concentrations. Indeed, proposed USEPA [7] regulations specifically take into account a measure of background EO concentrations in determining “delta c”, the facility-contributed concentration above the ambient background (the lowest measured fence line EO concentration subtracted from the highest measured fence line EO concentration). Thus, any risk-based action level also must account for background exposure. However, and importantly, these regulations do not consider total background EO exposure, which is composed not only of background concentrations inhaled from ambient air but also air equivalent exposures that everyone produces from normal metabolic processes (exogenous and endogenous background exposures) [1]. Background exposures, including endogenous levels in particular, provide an important reality check to inform the biological plausibility of dose-response models. The diminished attention to endogenous background exposures to EO by USEPA can be attributed to two reasons: (1) theoretical risk is defined in terms of extra exposure and risk above that posed by background exposure, and (2) the sterilization workers in the National Institute of Occupational Safety and Health (NIOSH) cohort that provided data for the USEPA assessment were exposed to EO concentrations (50,000->100,000 ppb) [8] that were orders of magnitude greater than typical background exposure. However, USEPA’s EO cancer risk assessment is guiding the regulation of the environmental exposures of populations residing near industrial facilities where the relationship between environmental EO exposure and agency risk-specific concentrations is dramatically different, i.e., total background exposures are indeed a trivial contribution to ppm-level occupational exposures to workers, but in contrast, facility-contributed low-ppb-level exposures to nearby residents are a small fraction of total background exposures.

Kirman and Hays [9] and more recently Kirman et al. [1] provided an analysis of background EO exposure. An alternative approach was initially developed for the calculation of total equivalent (TE, which includes endogenous and exogenous exposures) and endogenous equivalent (EE) background concentrations that allowed for the determination of the relative contributions of endogenous background, exogenous background, and EO-facility-contributed air concentrations to quantify near-facility population exposure [9]. The initial assessment of TE and EE values relied upon a meta-analysis for the biomarkers of EO exposure (specifically the hemoglobin adduct N-(2-hydroxyethyl)-valine or HEV) based upon published measurements in unexposed subjects. EE values resulting from this analysis ranged from 0.13 to 6.9 ppb for EO in air, which are orders of magnitude higher than the risk-specific concentration values derived for EO using USEPA’s revised cancer potency estimate. The calculation of TE and EE values for EO was then substantially updated to include new information: (1) HEV biomarker data collected by CDC as part of the National Health and Nutrition Examination Survey (NHANES); (2) a characterization of exogenous EO exposures using USEPA’s air monitoring data for EO; and (3) a characterization of the underlying biochemical pathways and sources of variation that contribute to endogenous exposures to EO [1]. TE and EE values resulting from the updated analysis ranged from 0.86 to 11.2 and 0.66 to 11 ppb, respectively, for EO in air. This background analysis showed that continuous background exposure concentrations are substantially greater than USEPA risk-specific concentrations (25,000 to 250 times greater than 10^−6^ to 10^−4^ risk-specific concentrations) and that the vast proportion of background EO exposure is associated with endogenous metabolism of ET to EO (~93%, endogenous exposure) rather than the smaller but measurable contribution from the inhalation of EO from ambient air (exogenous exposure). These background values have been shown to serve as a reality check when assessing the potential risks from EO exposures near chemical and sterilization facilities [1,10,11].

The quantitative characterization of U.S. nonsmoking population equivalent background exposures raised questions regarding the utility and effectiveness of the USEPA risk-specific concentrations in managing and communicating EO risk for populations residing near industrial facilities emitting EO to ambient air. These total and endogenous equivalent background EO exposure concentrations for the U.S. nonsmoking population provide a reality check on the health significance of near-facility-population total exposure concentrations. However, the USEPA [5] questioned aspects of the Kirman and Hays [9] and Kirman et al. [1] assessments of background EO exposures. USEPA specifically challenged the EO hemoglobin-adduct-based endogenous equivalent method as a valid biomarker for the reliable quantitation of total and endogenously generated EO exposures and for the measurement of low-level background exogenous EO exposures. It is therefore important to update and refine the overall background EO assessment based on the new data and information developed since the previous analyses and an enhanced validation of the total equivalent method coupled to the refined analytical measurements of EO background ambient air concentrations. Also, it is useful to assess how inferred assessment risk-specific concentrations from USEPA and TCEQ EO cancer risk assessments relate to U.S. population total background exposure as defined by the distribution of TE background exposure concentrations. Such a comparison of USEPA and TCEQ risk-specific concentrations to the TE concentrations for the U.S. nonsmoking population as characterized from this study should provide insight into the utility of these agency risk-specific concentrations in managing and communicating health risks for populations residing near industrial facilities.

The work presented here serves as an update to the previous TE and EE value derivations with specific consideration of the following: (1) new and corrected data are available from CDC NHANES on HEV burdens in U.S. nonsmokers and smokers; (2) new air monitoring data are available for EO in ambient air in the U.S. and near EO facilities; and (3) addressing questions regarding previously published TE and EE background assessments by using a refined validation of the EE exposure methodology based upon the direct analytical measurement of EO in the inhaled air of cigarette smokers that could then be used to independently validate the TE/EE-proposed linear relationship to low-level exogenous EO exposures. Each of these areas is summarized below.

### 1.1. New Data Are Available for HEV Burdens in the U.S.

In the assessment of Kirman et al. [1], two rounds of sampling for HEV burdens were available from CDC NHANES for U.S. nonsmokers and smokers (2013–2014 and 2015–2016), which included more than 4500 samples [12]. A new round of sampling data has been released (2017–2020), which includes more than 3500 additional samples [12]. In addition, CDC recently corrected the HEV data from all three rounds of sampling to address a systematic bias that was introduced due to purity issues associated with the calibration materials (personal communication with Dr. Benjamin Blount). As a result of this correction, the reported HEV values are now approximately 30% lower than reported previously. To avoid this situation in the future, the CDC has adjusted its policies and procedures with regards to the verification of calibration materials. The new and corrected data from CDC serve as the definitive dataset for characterizing total exposures (i.e., across all pathways) to EO in the U.S. and supersede the data used to support previous TE and EE derivations [1,9].

### 1.2. New Data Are Available for Exogenous Exposures to EO in Ambient Air

Nearly two years after the USEPA release of the EO reassessment, in late 2018, a number of state and local agencies began monitoring for EO at National Air Toxics Trends Stations (NATTS) and Urban Air Toxics Monitoring Program (UATMP) sites using EPA Method TO-15 for VOCs. In August 2019, USEPA in a Technical Note reported that EO had been officially added to the NATTS Tier 1 list for collection and analysis using EPA Method TO-15 [13]. EO concentration data from NATTS and UATMP programs are stored in the USEPA Air Quality System (AQS) online database. To date, full-year EO concentration measurements are available for 2019–2023. However, based on the method issues raised during this 5-year monitoring period, concentration data are not of equal quality throughout the period. Initially, questions were raised regarding the inadequacy of the TO-15 method detection limit given low ambient background concentrations. This is primarily an issue when a substantial fraction of samples is reported as below the method detection limit. To address this method detection limit issue, USEPA introduced a supplemental refined method for VOCs, Method TO-15A, in September 2019 to facilitate the collection and analysis of EO and other VOCs at lower concentrations [14]. Later, an observation of a positive concentration bias due to EO “growth”, unrelated to ambient EO concentration, was noted with some specific types of lined canisters. This EO growth on canister linings appeared to be exacerbated by holding samples for extended periods before analysis [15,16]. This positive growth bias was confirmed with controlled tests, and in May 2021, the USEPA issued a Technical Note that reported “canisters with a silicon-ceramic-lined inner surface appear to be less affected by the EO canister effect than those passivated with an electropolished inner surface for typical laboratory holding times (~30 days). Also, canister age and how thorough the canisters were cleaned before use were a factor in determining the extent of detectable EO concentrations” [17]. This Technical Note described stringent canister-clearing criteria and a canister blank certification process to mitigate lining growth and improve the representativeness of EO background measurements. The USEPA contract analytical laboratory also began identifying high biased concentrations (“LK” qualifier) among collected EO samples.

Based upon sensitivity and canister lining growth concerns, USEPA has questioned the quality and representativeness of earlier NATTS and UATMP monitoring results in characterizing ambient background EO concentrations. However, based on method refinements and stringent cleaning and certification criteria, it is reasonable to conclude that recent EO background concentration measurements made with Methods TO-15 and TO-15A and in accordance with the USEPA’s May 2021 Technical Note should be considered representative for statistically characterizing U.S. background EO concentrations. These observations concerning the methods related to early lower data quality and more recent higher data quality indicate a possible quality split within the five years of EO monitoring, with greater confidence in the quality of the recent three years. This study provides a comprehensive analysis of ambient background EO concentrations based on monitoring data from years 2019–2023, with a focus on 2021–2023 samples, a subset of NATTS samples collected with USEPA refined methods and canister cleaning criteria to characterize ambient background concentrations and exogenous background EO exposure with confidence.

### 1.3. Question Regarding Previously Published TE and EE Background Assessments

The derivation of EE values [1,9] has been questioned by USEPA [5]. Specific criticisms of the approach to calculating TE and EE values have included (1) a position that that the proposed linear relationship between HEV biomarkers and exposures to workers has not been factually substantiated for its application to low-level and background exposures to EO; (2) the EE analysis relies on the measurement of EO HEV adducts and does not include direct measurements of endogenous EO levels; (3) since cancer potency estimates for EO are expressed in terms of extra risk (i.e., above background), endogenous exposures are not included in the assessment; (4) other biomarker data (e.g., ET in exhaled breath) are not consistent with a large source of endogenous exposure; and (5) background ambient EO measurements away from emitting facilities are unreliable.

#### Smoker Validation

USEPA [5] suggested that the direct analytical measurement of EO in the inhaled air of smokers likely offered a reasonable set of low-level exogenous exposures that could be used to validate the TE/EE-proposed linear relationship between low EO exposures associated with background ambient air and smoking and the corollary formation of EO HEV adducts. Directly responsive to the USEPA recommendation, multiple published datasets were identified that characterized concentrations of EO in individual cigarettes that could then be converted to the total daily inhaled doses of EO in smokers dependent on the intensity of smoking behavior. This analysis shows a linear relationship between low EO exogenous exposures from ambient air and smoking and the corollary formation of EO HEV adducts that is consistent with the relationship observed with higher EO occupational exposures.

### 1.4. Study Objectives

To refine the population background EO exposure concentration estimates, facilitate the interpretation of the health significance of industrial EO exposure, and better inform the risk management decisions for populations residing near emitting facilities, the goals of this study are as follows:

- To summarize and incorporate the recent data regarding background exposures to EO to update the distribution of TE and EE concentrations for U.S. nonsmoking and smoking populations;
- To quantify the important sources of uncertainty and variation that may impact TE and EE values;
- To provide a characterization of the contribution of different exposures pathways contributing to total EO exposure in the U.S. population;
- To demonstrate the utility of the updated background TE and/or EE values in providing context to the health significance of EO exposures associated with populations residing near emitting industrial facilities compared to populations experiencing only background ambient and EE-contributed exposures;
- To show how risk-specific concentration values from USEPA and TCEQ cancer potency estimates relate to the distribution of background TE concentrations and affect the ability of these agencies to manage and communicate EO risk;
- To address select criticisms of previous TE and EE assessments, and particularly, the ability of the TE/EE method to reliably estimate total systemic EO exposures (endogenous plus exogenous).

This study is presented in two parts: (1) updating and refining the assessment of U.S. population background exposure to EO; and (2) applying the U.S. population distribution of background TE and EE exposure concentrations to provide context on the health significance of near-industrial-facility population EO exposures that are above background concentrations in ambient air.

## 2. Materials and Methods

Building upon our previous assessments [1,9] to characterize the potential exposures to EO within the U.S., we have relied upon the best available data from the published literature and from publicly available databases, as described below.

### 2.1. Derivation of Endogenous and Total EO Equivalent Exposures

**Figure 1:**
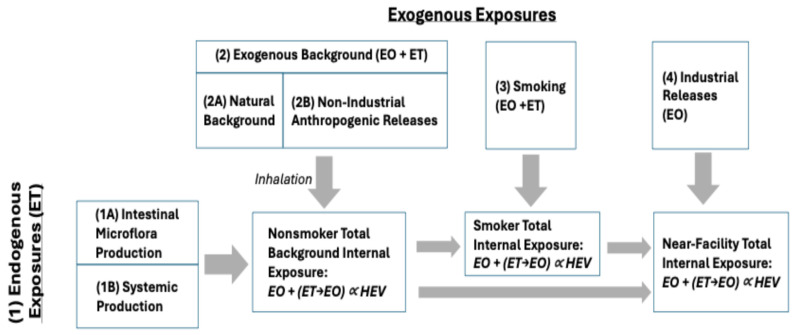
Conceptual model for exposure pathways contributing to total EO exposure based on HEV biomarker burden.

This section identifies the data and methods used to characterize exposures to EO in the U.S. and to apply TE and/or EE values as a reality check on the risk-specific concentrations calculated using USEPA and TCEQ cancer potency estimates. To support this updated assessment, a conceptual model of the exposure pathways that can contribute to total EO exposure was developed (Figure 1) and includes the consideration of the following pathway definitions:

- Endogenous Exposures: Internal exposure to EO that results from the production of ET by two general pathways, which is then metabolized to EO within the body (as summarized by Kirman et al. [1]): Pathway 1A: Production of ET by intestinal microflora; and Pathway 1B: Systemic production of ET (primarily hepatic). A small fraction of ET from both pathways is converted to EO within the body.
- Exogenous Exposures: Exogenous exposures to ET and EO arise from several possible pathways: Pathway 2A corresponds to the natural background of ET and EO in ambient air that results from natural sources of release (e.g., production of ET by plants; formation of ET and EO in wildfires); Pathway 2B corresponds to non-industrial anthropogenic releases of ET and EO (e.g., motor vehicle emissions, combustion of fuels) that can increase ambient air concentrations above natural background concentrations; Pathway 3 corresponds to smoking and serves as an important source of ET and EO; and Pathway 4 corresponds to industrial releases of EO that can result in ambient air concentrations that are above anthropogenic background concentrations. Potential exposures associated with the industrial release of ET were not considered in this assessment.
- Exogenous Background Exposure: Exogenous background exposures to EO reflect the combined contributions of ET and EO in air arising from the natural background (Pathway 2A) and anthropogenic, non-industrial releases (Pathway 2B). Monitoring data collected from rural and urban locations with no known industrial sources can be used to estimate the anthropogenic background.
- Total Background Exposure: Exposures reflecting the combined contributions from Pathways 1–3, which reflect typical exposures experienced by a nonsmoker or smoker who does not live near an industrial source.

#### 2.1.1. Characterization of Total and Endogenous Exposures to EO

HEV biomarker burdens measured in human populations reflect total exposure to EO via the exposure pathways conceptualized in Figure 1. In nonsmokers, HEV biomarker burdens can be described using the following equation:

*HEVTns* = *HEVE* + (*EOair* + *ETair* × *CF1*) × *CF2* (1)

where

- *HEVTns* = Total HEV biomarker burden in nonsmokers (pmol/g hemoglobin);
- *HEVE* = HEV biomarker burden attributed to the endogenous production of EO (pmol/g hemoglobin);
- *EOair* = Concentration of EO in air (ppb, continuous);
- *ETair* = Concentration of ET in air (ppb, continuous);
- *CF1* = Conversion factor to quantify the metabolic conversion of ET to EO within the body (unitless fraction);
- *CF2* = Conversion factor based on the correlation between exogenous exposures to EO and HEV biomarker burden (pmol/g per ppb continuous).

When rearranged, the equation can be used to solve the HEV burden associated with endogenous exposures:

*HEVE* = *HEVTns* − (*EOair* + *ETair* × *CF1*) × *CF2* (2)

The equivalent background air concentrations corresponding to HEVTns and HEVE burdens were calculated using the following equations:

*Total Equivalent* (*TE*) *Exposure* (*ppb*, *continuous*) = *HEVT*/*CF2* (3)

*Endogenous Equivalent* (*EE*) *Exposure* (*ppb*, *continuous*) = *HEVE*/*CF2* (4)

Monte Carlo simulations (1-dimensional) were used to incorporate the uncertainty and variation in parameter values to calculate the TE and EE values. Simulations were run for 10,000 iterations, and all parameters were assumed to be independent of one another (i.e., no correlations introduced). The text below summarizes the data and distributions used to support these simulations.

**Figure 2:**
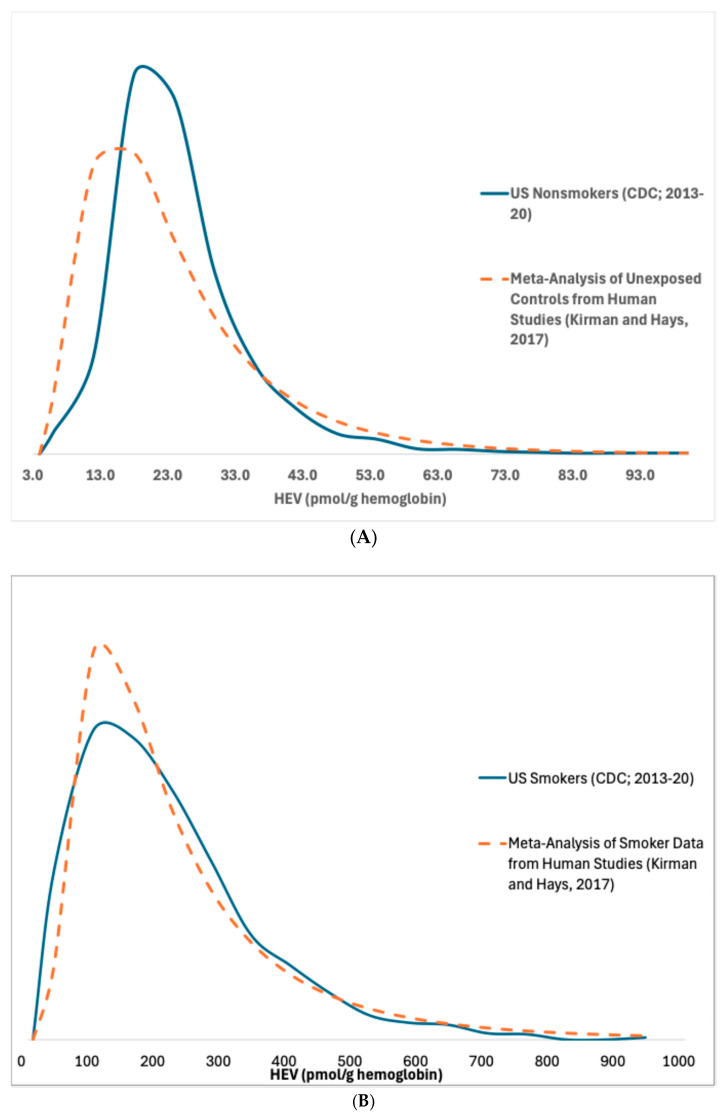
Total HEV in (**A**) nonsmokers and (**B**) smokers [9,12].

Additional calculations were performed to characterize the EO exposure Pathway 3 (from smoking), which is calculated as the difference between total HEV in smokers (HEVTs) and nonsmokers (HEVTns) (Figure 2):

*Smoking Total Equivalent Exposure* (*ppb*, *continuous*) = *HEVT_s_*/*CF2* (5)

Additional calculations were also performed to characterize EO exposure Pathway 4 (associated with industrial releases), calculated as the difference between EO exposures near facilities (EOair-f) and EO exposures for local background locations (EOair-bg):

*Site*-*Related EO Exposure* = *EOair*-*f* − *EOair*-*bg* (6)

*Site*-*Related HEV* = *Site*-*Related*
*EO*
*Exposure* × *CF2* (7)

#### 2.1.2. Characterization of Total Exposures to EO (Parameters HEVTns, HEVTs)

The U.S. Center for Disease Control has now collected and reported the biomarkers of EO exposure (HEV hemoglobin adducts) in the U.S. population over three sampling periods (2013–2014, 2015–2016, 2017–2020), which includes more than 8000 samples [12]. The bias-corrected biomarker data were used to characterize total exposures to EO, which includes Pathways 1, 2, and 4 for nonsmokers, and Pathways 1–4 for smokers (Figure 1). The NHANES data were analyzed and compared to previous characterizations of EO exposure in nonsmokers and smokers [9]. The parameters HEVTns and HEVTs were characterized using percentile values calculated for the NHANES dataset for nonsmokers and smokers, respectively, as summarized in Table 1.

#### 2.1.3. Characterization of Exogenous Exposures to EO and ET

##### Exogenous Background Exposures to EO

EO concentrations in ambient air are available from national ambient EO monitoring data collected under the USEPA NATTS and UATMP monitoring programs between late 2018 and 2023. EO concentration data from the USEPA monitoring sites were queried from the USEPA AQS database [18]. Monitoring data were collected beginning in October 2018 (*n* = 157 measurements in 9 states); however, our analysis focused on the full-year collections between 2019 and 2023. The raw ambient EO monitoring data collected and reported by USEPA between January 2019 and December 2023 available for the analysis of ambient background concentrations are described in Table 2.

These data generally reflect exogenous background exposures (natural background plus non-industrial anthropogenic releases; Pathway 2 in Figure 1). The number of states and site locations at which EO monitoring data were available varied by year and ranged from 11 states (in 2019) to 22 states (in 2023) and from 26 site locations (in 2019) to 61 site locations (in 2021). Across the various site locations, samples were collected on average every 3 to 14 days. All samples were long-term (24 h) and collected via EPA Compendium Method TO-15 (hereafter as “passive”) or TO-15A (hereafter as “pressurized”) collection methods, both of which were incorporated into the NATTS Technical Assistance Document. The frequency of non-detect samples (defined as concentrations reported as 0 ppb) ranged from 3.0% to 18.9% across years and collection methods. Statistical analyses showed that the mean concentration for the early monitoring years 2019 and 2020 are significantly greater than the mean concentrations for the later monitoring years 2021–2023. This finding is consistent with the period prior to method refinements (pre-refinement) and the period following method refinements to minimize bias (post-refinement), described previously. Therefore, the focus of the TE and EE analyses relies upon the combined concentration data from the years 2021–2023 that are considered generally free of bias and representative of ambient background concentrations, and therefore reflective of background exogenous exposure.

The ambient monitoring data summarized in Table 2 present the data as directly available from the USEPA AQS database, which provides data in the manner it is reported by each state (i.e., represents a “raw” dataset). Therefore, we conducted several data cleaning steps prior to performing our analyses. Potential outliers (defined as data more than three standard deviations above mean concentrations, i.e., Z-score +/− 3) were removed from the dataset (EO > 0.68 ppb, n = 154 measurements). Non-detect data were assigned a value of limit of detection divided by 2 (0.015 ppb; n = 1480 measurements). In a sensitivity analysis, site locations with greater than 20% non-detect data were removed; however, the modified dataset was determined to be statistically indistinguishable from the full dataset, and thus these site locations were retained in the main analysis. Finally, to provide the most current and reliable statistical characterization of ambient background EO levels, data for which the “LK” qualifier was flagged were removed from the dataset (n = 2968 measurements). This final dataset is referred to as the “EO clean dataset” throughout this manuscript. The EO clean dataset was examined by year and collection method; for each year and collection method, the mean and standard deviation (SD) were calculated, along with the 5th, 10th, 25th, 75th, 90th, and 95th percentiles. Differences in mean concentrations by collection method and year were examined using *t*-tests. Given the right-skewed nature of these data, comparisons were performed using *t*-tests with log-transformed data. Mean concentrations by year were compared to assess statistically significant differences and the two different groups consistent with the pre-refinement and post-refinement monitoring conditions. Samples within these two groups were then combined and statistically analyzed to characterized representative EO concentrations for the monitoring period before sampling method refinements were implemented (2019–2020) and the period following the implementation of those method refinements.

##### Characterization of Exogenous Exposures to ET

Health Canada and the University of Windsor collected 24 h personal, indoor, and outdoor exposure samples for a large number of non-polar volatile organic compounds (VOCs) including ET [19]. Given the proximity of the study location to a major U.S. city (Detroit, MI), this included study is considered to be representative of potential exposures in the U.S. as well. These data are considered to reflect exogenous background exposures to ET (natural background plus non-industrial anthropogenic releases; Pathway 2 in Figure 1). The parameter *ETair* was defined using summary statistics provided by this study (Table 3).

#### 2.1.4. Conversion Factors

For the metabolic conversion of ET to EO within the body (parameter CF1), the central tendency estimate was defined by predictions made by the PBPK model of Filser and Klein [20] in humans, and an upper-bound estimate was defined by the value reported by Csanady et al. [21] (Table 3). To characterize the relationship between exogenous exposures to EO and HEV burden (parameter CF2), a robust standard errors linear regression analysis was performed on data from studies reporting HEV measurements in workers with known exposures to EO [21,23,24,25,26,27,28,29,30,31,32] (Table 3). Many of these datasets have been used previously to characterize the relationship between occupational exposure to EO and measured HEV burdens [20,21]. To support the application of the HEV correlation in exposed workers to lower exposure levels (e.g., ambient air exposures to EO), the results of the worker regression analyses were compared to previously published linear relationships and to additional linear regression analyses of NHANES HEV data collected from U.S. smokers and nonsmokers.

#### 2.1.5. Independent Validation of a Low-Dose Linear Relationship for HEV and EO Exposure Based on Smoker Data

Multiple published datasets were identified that characterized concentrations of EO in individual cigarettes that could then be converted to the total daily inhaled doses of EO in smokers exposure dependent on the intensity of smoking behavior [33,34,35,36]. To convert between smoking exposures (cigarettes per day) and the equivalent exposure in air (ppb continuous), the following approach was used. Liu et al. [33] reported for Kentucky Reference 3R4F cigarettes mean concentrations of 8.37 μg EO/cig under the International Organization for Standardization (ISO) smoking regimen and 26.03 μg EO/cig under the “Health Canada intensive” (HCI) smoking regimen. Forster et al. [34] reported for the updated Kentucky Reference 1R6F cigarettes mean concentrations of 17.2 μg EO/cig (HCI) and 19.3 μg EO/cig (HCI) for Kentucky Reference 3R4F cigarettes. Jaccard et al. [36] reported for Kentucky Reference 1R6F cigarettes mean concentrations of 5.92 μg EO/cig (ISO) and 17.3 μg EO/cig (HCI). Jaccard et al. [36] also reported for Kentucky Reference 3R4F cigarettes, which yielded mean concentrations of 6.78 μg EO/cig (ISO) and 19.2 μg EO/cig (HCI). Equivalent EO exposure concentrations in air (EC) can be estimated as C × CpD/IR, where C is the reported EO concentration per cigarette (μg/cig), CpD is the number of cigarettes smoked per day, and IR is the daily inhalation rate (m^3^/day). CpD is conservatively assumed to be 17 CPD based on the average number of cigarettes smoked by daily smokers in 2005, as reported by CDC [37], and IR is assumed to be 16 m^3^/day based on the mean inhalation rates for adults aged > 16 yrs [38]. These data were used to support the comparison of the linear regression results for slope for data with different exposure metrics (ppb in air, CpD).

#### 2.1.6. Software

The summary statistics and calculations were performed in Microsoft Excel (version 16.83). Monte Carlo simulations were performed using the open-source Excel add-in XLRisk (https://github.com/pyscripter/XLRisk; accessed on 5 October 2023). Linear regressions were performed in R using the packages “tidyverse” and “sandwich”. Given the comparatively small sample size of the HEV worker data, robust standard error methods (HC3) were used to provide more accurate, unbiased, and efficient estimates of the standard errors for the regression coefficients. This method also results in a larger SE value than standard linear regression and was used to characterize parameter CF2 to avoid underestimating this source of variation. For the analysis of NHANES HEV data, which has a large sample size, standard linear regression methods were used.

### 2.2. Application of Endogenous and Total Equivalent Exposure Estimates to Assess Exposures near EO Facilities

#### 2.2.1. Characterization of Excess Exogenous EO Exposures near Industrial Facilities

EO concentrations from recent monitoring programs near emitting facilities in Georgia and Utah and representative background locations are evaluated to provide context for exposures experienced by populations residing near these facilities (Table 4). For Georgia, 2021 and 2022 24 h annual and partial annual EO sample concentrations from six monitoring locations adjacent to a Sterigenics facility in Smyrna, five locations adjacent to a Becton Dickenson (BD) facility in Covington, and three locations adjacent to a Sterilization Services facility in Atlanta, along with background reference locations in General Coffee Park and South DeKalb, were analyzed to provide context. At all near-facility locations and at the General Coffee Park background location, only passive samples were collected. In contrast, both passive and pressurized samples were collected at the South DeKalb commercial area background location. Similarly for Utah, 24 h 2022 seasonal EO passive samples collected from eight residential locations near a BD Medical facility and four commercial locations near a Sterigenics facility in Salt Lake County, as well as five background reference locations, were analyzed. The samples from both the Georgia and Utah locations were collected following USEPA methods intended to maximize sensitivity and minimize bias. In addition, the USEPA contract laboratory analyzing the samples in these studies identified sample concentrations meeting the criteria of being biased high. Samples that qualified as LK (biased high) were removed from the analyses.

Prior to the analysis of these data, we assigned the monitoring data reported as ND with a value of method detection limit divided by 2, and averaged the concentration taken for QA pairs. Also, for background samples, outliers and LK biased high concentrations were removed from the statistical calculations. The mean, median, and standard deviation (SD) of the EO concentrations were calculated for each monitored site by year or monthly period within a year (or season [summer and winter months] for Utah samples). To provide the initial context of exposure significance, a statistical comparison of exogenous background and near-facility EO concentrations was performed (i.e., are facility emissions producing ambient concentrations significantly greater than the exogenous background concentrations?). Given the skewed nature of these data, the comparisons of concentrations for matched date ranges were performed using *t*-tests with log-transformed data and an additional non-parametric test, a Wilcoxon rank-sum test of medians. To adjust for multiple comparisons and, therefore, minimize the potential for a statistically significant result due to chance, a Bonferroni correction was performed to the standard alpha of 0.05, resulting in a more stringent significance criterion (e.g., for Georgia comparisons, 0.05 divided by 21 comparisons = 0.0024). As the Georgia background locations differed in sample type composition, near-facility location mean/median sample concentrations were compared to the General Coffee Park and South DeKalb background location mean/median sample concentrations separately. Utah seasonal near-source mean/median sample concentrations were compared to an aggregate mean/median concentration for the five background locations, as there was only a statistically significant concentration difference between two of the five background locations. These data were used to define the parameter EOair to characterize exposure Pathway 4 (Figure 1) for the purpose of comparing to other exposure pathways and performing reality checks for risk-specific concentration values.

#### 2.2.2. Comparison of Near-Facility Population Total Exposure Concentration to Background TE Concentrations

An additional reality check for population exogenous exposure to near-facility concentrations significantly greater than the ambient background is provided by comparing population total EO concentration exposure with total equivalent (TE) U.S. population background exposure [i.e., how does their continuous total exposure concentration from endogenous and exogenous sources compare to the distribution of continuous total equivalent background EO exposure concentration (endogenous and exogenous) for the nonsmoking U.S. population?]. This comparison can be evaluated in two ways: (1) the estimated near-facility residential population EO total continuous exposure concentration (50th percentile endogenous background concentration from all ET sources + 50th percentile exogenous exposure concentration) compared to the distribution of the total equivalent EO concentrations for the nonsmoking U.S. population based on current analyses, or (2) the near-facility population exogenous exposure concentration that is above the background exogenous concentration + 50th percentile total equivalent exposure concentration compared to the normal distribution of total equivalent exposure concentrations for the nonsmoking U.S. population based on current analyses.

## 3. Results

The results of this study are presented in two parts: (1) an updated characterization of TE and EE continuous background EO exposure concentrations for the nonsmoking and smoking U.S. populations away from industrial sources and (2) the application of the distribution of TE and/or EE background concentrations as a reality check on the health significance of excessive exposure to EO in ambient air that U.S. populations residing near emitting facilities potentially receive.

### 3.1. Endogenous and Total Equivalent Exposures

#### 3.1.1. Total Exposures to EO

Frequency distributions for HEV measurements in U.S. smokers and nonsmokers from NHANES [12] are provided in Figure 2. The distributions based upon the NHANES updated and corrected data compare well with distributions published previously based upon a meta-analysis of studies of HEV measurements in nonsmokers and smokers [9]. The NHANES data indicate that, on average, HEV levels in smokers are approximately 8-fold higher compared to nonsmokers (respective arithmetic means of 179 and 23 pmol/g) (Table 1). Due to CDC’s recent bias correction of the raw HEV data, these arithmetic mean values calculated here are approximately 30% lower than those calculated previously based on NHANES 2013–16 data before the bias correction (respective means of 236 and 31.4 pmol/g in smokers and nonsmokers; Kirman et al. [1].

#### 3.1.2. Background Exogenous Exposures to Ambient EO Concentrations in the U.S., EOair

A summary of the monitoring data in the clean EO dataset, by year, pre-and post-refinement groups, and collection method, is shown in Table 5.

For data collected using the passive collection method, the mean concentration of the 2019 and 2020 data was determined to be statistically significantly different from the data collected all other years (*p* < 0.05), while the mean concentrations of the 2021–2023 data were found to be statistically indistinguishable from one another. For data collected using the pressurized collection method, the mean concentration of the 2019 and 2020 data was similarly determined to be statistically significantly different from recent years; however, the mean concentration of the 2023 data was statistically significantly different from the 2021 and 2022 means. Based on the statistical similarity in the 2019–2020 and 2021–2023 concentrations and the consistency of the dates of these two groups with the implementation date of the method refinements, the concentrations from these year groups were combined and analyzed to provide a statistical characterization of pre- and post-method refinement ambient background EO concentrations. The mean concentrations for the combined pre-refinement 2019–2020 data and the combined post-refinement 2021–2023 data are statistically significantly different for both the passive and pressurized samples in these groups. The pre-refinement and post-refinement mean background ambient EO concentrations for the passive and pressurized method collections are 0.088 and 0.076 and 0.066 and 0.072 ppb, respectively. The post-refinement EO concentration years 2021–2023 are considered here to be representative of the typical exogenous background EO concentrations experienced by the U.S. population. The median pre-refinement passive and pressurized concentrations (0.05 and 0.065 ppb), respectively, are within the low-end range of historical (1989–2016) median EO concentrations (0.04–0.15 ppb) [45], suggesting that sampling method issues are the primary reason for differences with the representative post-refinement concentrations.

#### 3.1.3. Relationship Between Exogenous EO Exposures and HEV Biomarker Burdens (Parameter CF2)

##### Linear Regression Analysis of Worker HEV Data

**Figure 3:**
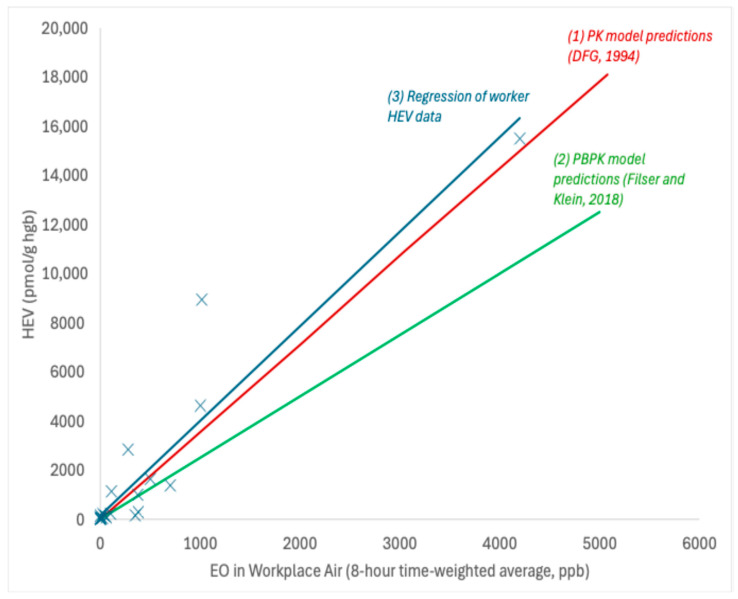
Relationship between HEV and occupational exposures to EO; X = data point for HEV in workers with known EO exposure; solid blue line = robust SE linear regression; solid red line = PK model predictions [22]; solid green line = PBPK model predictions [20].

HEV data collected in exposed workers across multiple studies [23,24,25,26,27,28,29,30,31,32] provide support for a linear relationship between EO in workplace air (measured air concentrations range from ~8 to 4200 ppb occupation exposure) and the measured HEV biomarker burdens (measured HEV range from ~45 to 15,000 pmol/g; Figure 3). A robust SE linear regression analysis of these data (solid blue line in Figure 3) yields a slope of 3.9 ± 1.4 pmol/g per ppb occupational exposure, which corresponds to a slope of 11.3 ± 4.2 pmol/g per ppb continuous exposure (assuming breathing rates of 10 and 20 m^3^/day and exposure frequencies of 250 and 365 days/year for worker and continuous exposures, respectively). The slope value based on worker HEV data for EO is very similar to those predicted by the pharmacokinetic model of DFG [22] (approximately 3.6 pmol/g per ppb occupational) and the PBPK model of Filser and Klein [20] for EO (approximately 2.5 pmol/g per ppb occupational), both of which fall within one standard error of the slope defined by the worker HEV regression. The slope value from this linear regression analysis is also very similar to the slope used in previous TE and EE assessments [1,9] (11.3 vs. 10.9 pmol/g per ppb).

##### Linear Regression Analysis of HEV Data from NHANES for Nonsmokers and Smokers

**Figure 4:**
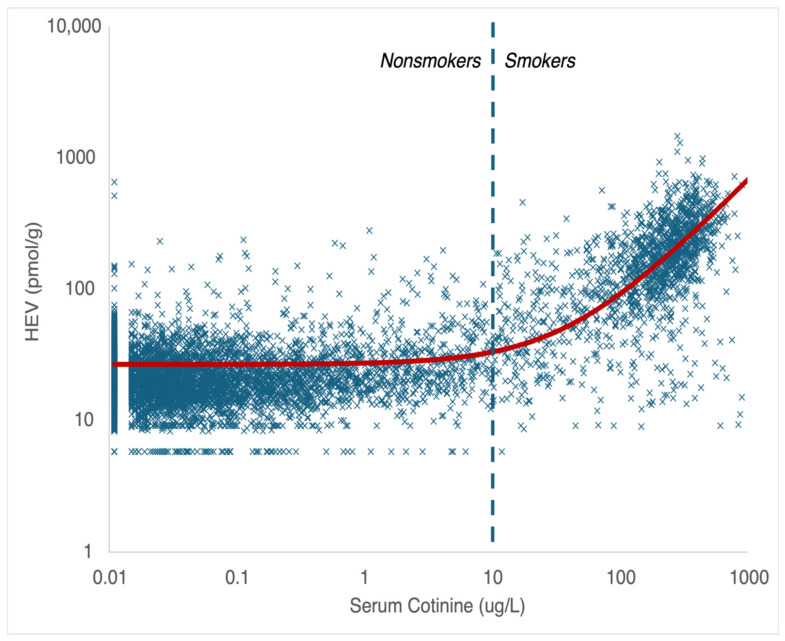
HEV as a function of serum cotinine (NHANES data; 2013–2020); X = data point; solid red line = linear regression; dashed blue line = upper limit for serum cotinine in nonsmokers.

**Figure 5:**
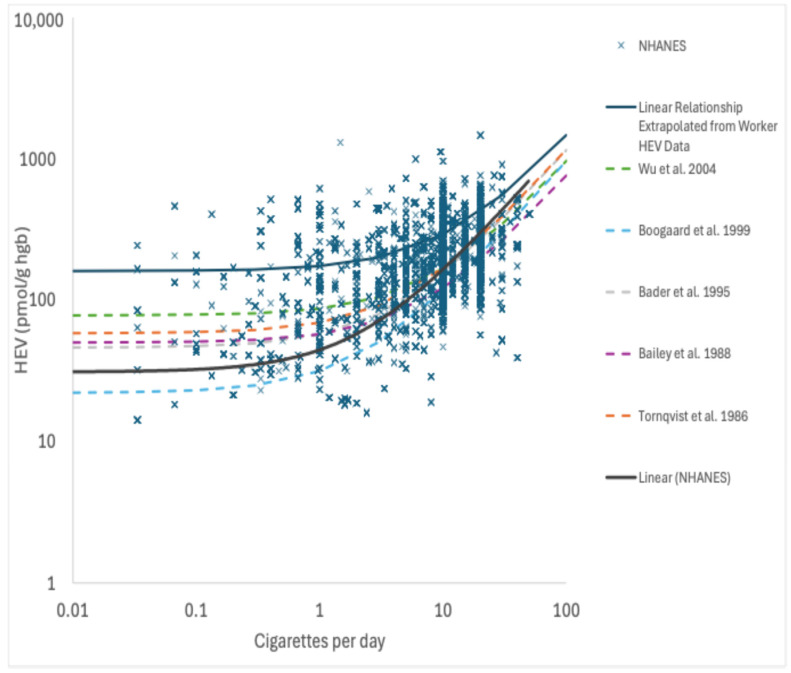
HEV as a function of cigarettes per day (NHANES data; 2013-2020); X = data point; solid blue line = linear regression of NHANES data; solid red line = linear regression prediction from worker HEV data extrapolated from Figure 3; dashed lines = reported relationships for HEV and CPD from the published literature sources. References as cited in main text: Wu et al. 2004 [46], Boogaard et al. 1999 [25], Bader et al. 1995 [47], Bailey et al. 1988 [48], and Tornquist et al. 1986 [49].

The linear regression analysis of the HEV data on nonsmokers and smokers collected as part of NHANES as a function of serum cotinine, which serves as a biomarker of exposure to tobacco smoke (a source of EO exposure), yields a linear relationship across a wide range of exposures that spans five orders of magnitude (measured serum cotinine range of approximately 0.011–1000 μg/L; measured HEV range of approximately 5.8–1500 pmol/g), as indicated by the solid red line in Figure 4 (note—the log-log scale utilized in this figure to permit inspection of the data behavior at low exposures gives this line a nonlinear appearance). The upper end of the range for measured HEV values in smokers in Figure 4 shares considerable overlap with the lower end of the range of measured HEV values in workers in Figure 3. An additional linear regression analysis of the HEV data for smokers as a function of their self-reported average cigarettes per day (CpD; for the previous 30 days) also yields a linear relationship with a slope of 13 pmol/g per CpD (Figure 5). This value is similar to but slightly higher than the linear relationships reported for CpD and HEV in the published literature (7.1–11 pmol/g per CpD; [46,47,48,49]). Furthermore, extrapolating the linear relationship defined by the worker HEV data (Figure 5) also yields a similar slope (within a factor of approximately 2; 11.3 pmol/g per ppb continuous × 0.53 = 6.0 pmol/g per CpD) but with a different y-intercept (Figure 5; note—since the worker HEV data were collected from higher exposures compared to smoker exposures, they are not particularly informative on the y-intercept value, as indicated by the relatively large SE value for this term). There is no evidence to support nonlinear toxicokinetic behavior for EO with the data depicted in Figure 4 and Figure 5. As such, these data and analyses indicate that the direct measurement of EO in cigarette smoke indicates that EO exposures contributed by smoking support a continuous linear relationship between low smoking up to high occupational exogenous EO exposures and EO HEV adduct biomarker levels.

#### 3.1.4. TE Values, EE Values, and Pathway Contributions to Total EO Exposure

Distributions for the HEV biomarker burdens attributed to endogenous exposure (from Pathways 1 and 2 from Figure 1) and the corresponding TE and EE values for different statistics for HEVT and HEVE are provided in Table 6. The equivalent air concentration corresponding to the mean endogenous HEV level 3.3 ppb (90% CI = 1.1–5.0 ppb) is similar to the previously published value (2.7 ppb) [1]. The equivalent air concentration corresponding to the standard deviation for endogenous HEV level 2.5 ppb (90% CI = 0.8–3.9 ppb) is also similar to the previously published SD value (2.3 ppb) [1]. Percentiles (1–99%) for EE and TE values range from approximately 0.7 to 11.5 ppb and 0.9 to 11.7 ppb, respectively.

**Figure 6:**
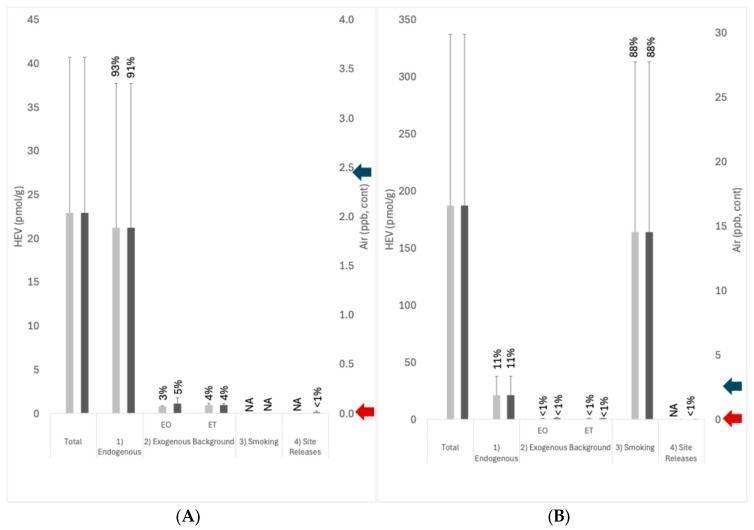
Pathway contribution to total HEV burden for (**A**) nonsmokers and (**B**) smokers; light gray columns indicate exposures remote from EO facilities; dark gray columns indicate exposures near EO facilities; error bars = standard deviation; percent values indicate the percentage of total exposure attributed to the pathways; blue arrow on the right vertical axis indicates the 1 × 10^−5^ risk-specific concentration based on TCEQ cancer potency (2.4 ppb); red arrow on the right vertical axis indicates the 1 × 10^−5^ risk-specific concentration based on USEPA cancer potency (0.0011 ppb). Exogenous background exposures (Pathway 2) were characterized using national air monitoring data for remote-facility exposures and using local air monitoring data for near-facility exposures. Exposures attributed to facility-related site release (~0.11 ppb) were estimated using the arithmetic mean for Steriogenics sample locations S6 and S7 in 2021–2022 less the arithmetic mean calculated for the local background.

The results for the contributions of each of the four exposure pathways identified in the conceptual model (Figure 1) to total EO exposures are summarized in Figure 6. For U.S. nonsmokers that are not living near EO facilities, endogenous exposures (Pathway 1) on average account for a majority (~93%) of total background exposure to EO, whereas exogenous background exposures (Pathway 2) account for ~7% of total exposure (~3% from exogenous EO, ~4% from exogenous ET) (Figure 6A). For U.S. smokers that are not living near EO facilities, smoking (Pathway 3) on average accounts for a majority (~88%) of total background exposure, and endogenous exposures (Pathway 1) account for ~11% of the total exposure to EO, while exogenous background exposures (Pathway 2) account for <1% of total exposure (~0.4% from exogenous EO, ~0.5% from exogenous ET) (Figure 6B). The pathway contributions for both nonsmokers and smokers are very similar to those estimated previously [1].

For U.S. nonsmokers and smokers who live near EO facilities, total EO exposures are slightly higher (by 3% and <1%, respectively) over those who live remotely from facilities (Figure 6). Most of this difference is attributed to local background concentrations of EO that are slightly higher than the national background concentrations. For U.S. nonsmokers living near EO facilities, endogenous exposures (Pathway 1) on average account for a majority (~91%) of total exposure to EO, exogenous background exposures (Pathway 2) account for ~9% of total exposure (~5% from exogenous EO, ~4% from exogenous ET), and exposures associated with facility releases (Pathway 4) account for ~ < 1% of total exposure (Figure 6A). For U.S. smokers living near EO facilities, smoking (Pathway 3) on average accounts for ~88% of total exposure, endogenous exposures (Pathway 1) account for ~11% of total exposure to EO, anthropogenic background exposures (Pathway 2) account for <1% of total exposure, and exposures associated with facility releases account for <1% of total exposure (Figure 6B).

When viewed in isolation, a comparison of the added exposures associated with site releases (Pathway 4 in Figure 6; ~0.11 ppb) to USEPA’s 1 × 10^−5^ risk-specific concentration of 0.0011 ppb would lead risk assessors/managers to a conclusion of there being an unreasonable risk to human populations living near EO facilities. In contrast, a comparison of these exposures to TCEQ’s 1 × 10^−5^ risk-specific concentration of 2.4 ppb leads to the conclusion that the potential risks to these populations are below levels of concern. Furthermore, placing these exposures within the context of total exposures to EO experienced by the U.S. population from Pathways 1–3 leads to a conclusion that the potential exposures to these populations from site releases are not biologically meaningful both in terms of their magnitude (less than 1 percent of total exposure) and normal variation (less than 1 percent of one standard deviation for total exposure).

#### 3.1.5. Smoker Equivalent Exposures to EO

As noted above, smokers experience exposures to EO that are 8-fold higher than nonsmokers on the basis of their HEV burdens. The total EO exposures to U.S. smokers is equivalent on average to continuous exposure to an air concentration of 16.6 ppb with a range of 1.2–58.4 ppb based upon the 1st-99th percentile (Table 7).

#### 3.1.6. Independent Estimate of Smoking Exposure Equivalents for EO

The estimated ECs for EO from the validation analysis ranged from 3.5 to 15 ppb (arithmetic mean of 8.9 ± 4.3 ppb). Based upon calculations, a conversion factor for equivalent smoking exposures of 0.53 ppb continuous per CpD (~8.9 ppb/~17 CpD) was derived.

### 3.2. Application of Endogenous and Total Exposure Estimates to Assess Exposures near EO Facilities

#### 3.2.1. Risk Management and Risk Communication Context for Near-Facility Potential Population Exposure Concentrations

The background ambient EO concentrations (background exogenous exposure concentrations) and background total exposure concentration (exogenous plus endogenous, EE) characterized as TE continuous background concentrations provide two levels of context as to the health significance of excessive ambient EO concentrations above ambient concentrations for populations residing near industrial facilities.

##### Comparisons to Background

To provide an initial level of context, EO concentrations from monitoring sites near emitting facilities are statistically compared to representative local, regional, or national background concentrations to identify concentrations at locations elevated above the background (i.e., additional industry-related exogenous exposures). A summary of recent monitoring, statistical characterization of EO concentrations, and comparison of near-facility and background concentrations of three Georgia facilities is presented in Table 8.

One of the first things to note regarding the Georgia background locations is that there is no significant difference between the mean concentrations from the passive samples in 2021 and 2022 from the General Coffee Park location and the 2021–2022 passive and pressurized samples from South Dekalb. However, there is a statistically significant difference in mean EO concentration between the 2021 General Coffee Park and South Dekalb passive samples. The significant difference in samples of the same type from the two background locations indicates that they should be treated independently. Therefore, near-facility location concentration comparisons were performed separately with samples from the General Coffee Park and South DeKalb background locations as well as from U.S. background concentrations. It is not surprising that the 2021 50th percentile passive background sample concentration from General Coffee Park was consistent with the 2021 U.S. 50th percentile passive background sample concentration (0.051 to 0.050 ppb), as was the 2022 mean pressurized background sample concentration from South Dekalb with the 2022 U.S. mean pressurized background sample concentration (0.068 to 0.070 ppb).

The mean EO concentrations from five of the fourteen near-facility monitoring locations (S3, S6, F1, F2, and C2) were significantly greater than the mean concentration from the General Coffee Park background location based on the 2021 samples, while only the mean concentrations from two of seven monitoring locations (C2 and F2) exceeded the General Coffee Park mean background concentration based on the 2022 samples. Only the 2021 sample mean concentrations from locations S3 and F2 were significantly greater than the mean concentration for the South Dekalb background samples, and only the 2022 mean concentration from location F2 significantly exceeded the South Dekalb background mean concentration. At this location, the near-facility mean EO concentrations in 2021 and 2022 were ~2.4 times the mean background concentration. An additional comparison with U.S. passive background and near-facility concentrations identified the mean concentrations from three additional near-facility locations in 2021 (S2, S7, and C7) as being significantly greater than the national background mean concentration. We note that local background concentrations are superior to the national background concentration when evaluating statistical differences considering local meteorological conditions. However, the state or national background may provide utility in these comparisons when no local background data are available or where a representative local background ambient concentration is difficult to collect. A similar summary of recent monitoring, statistical characterization of EO concentrations, and comparison of near-facility and background concentrations for two Utah facilities is presented in Table 9.

Recent near-facility and background concentrations in Utah were collected for a few months in the summer and winter, unlike the annual or longer-term collections described above for Georgia. The mean summer background concentration was significantly greater than the mean winter background concentration. However, these seasonal near-facility and background concentration data support summer and winter seasonal statistical comparisons. Mean summer background EO concentrations from all four locations were statistically indistinguishable based on the ANOVA test log-transformed data and the non-parametric Kruskal–Wallis test. The mean winter background EO concentrations from the five locations were also statistically indistinguishable based on the ANOVA test; however, the mean winter EO concentrations for locations BG4 and BG5 were significantly different based on the non-parametric Kruskal–Wallis test. However, for comparisons of background concentrations with near-facility concentrations, both the summer and winter background mean EO concentrations are from the combined seasonal observations from all locations and assumed to fit a log-normal distribution.

During warm weather summer conditions, the mean EO concentrations for six near-facility locations (BD1, BD2, BD3, SG1, SG2, and SG4) were statistically greater than the mean summer background concentration. For the cold weather winter period, only the mean concentrations for BD1 and SG1 were greater than the mean background concentration. There were dramatically higher near-facility and background EO concentrations under warm weather summer conditions than cold weather winter conditions. This seasonal difference was also obvious in Georgia EO concentrations.

##### Total Exposure and Total Equivalent Background Exposure Concentration Comparisons

Based on the analyses presented, we note that everyone is exposed to background concentrations of EO unrelated to industrial emissions and that most of everyone’s daily exposure is endogenous from the metabolism of ET rather than exogenous from the inhalation of EO in ambient air. Exogenous EO exposure contributes only a small fraction of the U.S. population continuous total EO background exposure (~3%). Thus, to provide a reality check and context on the health significance of near-facility population exposure when mean near-facility ambient EO concentrations statistically exceeded mean ambient background EO concentrations, total population EO exposure concentration should be considered. This can be achieved by determining if total exposures are outside the normal background exposure range, specifically, comparing the 50th percentile TE background concentration plus the portion of near-facility 50th percentile ambient concentration above the background with the distribution of the background TE or EE (as it composes 93% of TE) EO concentrations for the nonsmoking U.S. population based on current analyses.

**Figure 7:**
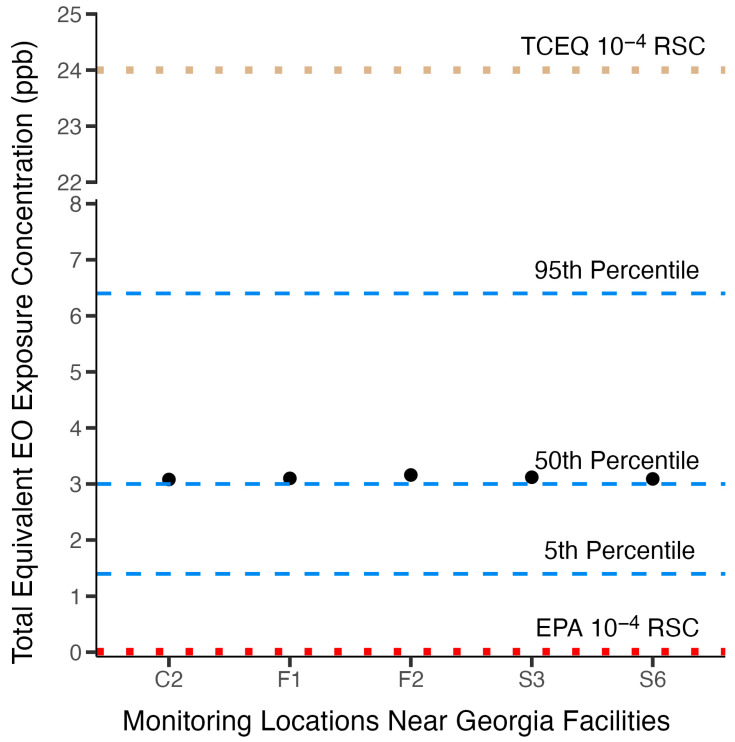
Near-facility potential total EO continuous exposure concentrations (black dots) are plotted against the 5th, 50th, and 95th percentile total equivalent background exposure concentrations and USEPA and TCEQ 10^−4^ risk-specific concentrations.

Figure 7 shows the potential total exposure concentrations for populations residing near facility locations in Georgia, where annual ambient EO concentrations exceeded annual ambient background concentrations, and how these total population EO exposure concentrations relate to the distribution of TE background concentrations for the U.S. nonsmoking population. It is clear that the added concentrations from facility emissions at these locations contribute little to increase population exposure above the 50th percentile total equivalent background concentration for the U.S. nonsmoking population. The total near-facility population exposures are well below the 60th percentile TE (3.3 ppb). This observation is consistent with the analyses that show that exogenous EO exposure contributes only a small fraction (~3%) of total U.S. population background exposure, and therefore facility excess concentrations above the background have little effect on population total exposure. Starting from the 50th percentile TE concentration, it would take a near-facility increase in ambient concentration above the background by more than 3.4 ppb to reach the 95th percentile TE background concentration or more than double the 50th percentile TE background concentration to reach an upper bound of the normal background range.

The exogenous EO concentration measurements from the Utah study, both near-facility and background, were collected for only ~ two months during winter and summer weather conditions and as such cannot be normalized to annual mean concentrations for these monitoring locations. In this respect, they are dissimilar with the annual background and near-facility exogenous concentrations from the Georgia facility studies. Therefore, these limited seasonal concentrations are not directly amenable to estimates of near potential facility population total exposure concentrations (ambient and background concentrations) to compare with U.S. population continuous annual total equivalent background exposure concentrations as described above for Georgia near-facility populations. However, for the Utah study, such a comparison of the largest exceedances from the background for near-facility locations for each season, assuming that seasonal means represent annual means, could provide a conservative reality check of exposure significance. Near-facility summer concentrations above the exogenous background at locations BD1 (0.28 ppb) and SG2 (0.29 ppb) would contribute to the total population concentration exposures for BD1 (3.28 ppb) and SG2 (3.29 ppb), which are approaching the 60th percentile total equivalent exposure concentration (3.3 ppb) for the U.S. nonsmoking population. Near-facility winter concentrations above the exogenous background at locations BD1 (0.077 ppb) and SG1 (0.071 ppb) would contribute to the total population concentration exposures for BD1 (3.08 ppb) and SG1 (3.07 ppb) levels just above the 50th percentile total exposure concentration (3.0 ppb). This conservative comparison illustrates that even assuming that short-term high near-facility exogenous concentrations at ~four times the annual background are representative of continuous annual ambient exposure concentrations, it has a negligible effect on total EO exposure, which remains well within the center of the normal population background range.

##### Consistency of Background Exposure Concentrations with Risk-Specific Concentrations Inferred from USEPA and TCEQ Risk Assessments

As described in detail above, a major reason for the difference between the USEPA [2] and TCEQ [3] inhalation cancer risk assessments for EO is that the risk-specific concentrations are derived using different and divergent dose-response models. These different dose-response models produced substantially different risk-specific concentrations. The 10^−6^ to 10^−4^ risk-specific concentrations calculated from the USEPA and TCEQ cancer potency values are 0.00011–0.011 ppb and 0.24–24 ppb, respectively. It is illustrative to see how the RSCs, based on extra risk, from these two risk assessments relate to the total equivalent background exposure concentrations for the nonsmoking U.S. population. Figure 7 shows the 10^−4^ risk-specific concentrations from the USEPA and TCEQ assessments relate to the 5th, 50th, and 95th percentile total equivalent background continuous exposure concentrations for the U.S. nonsmoking population. It is readily apparent from this figure that the USEPA high-risk-range risk-specific concentration value (0.011 ppb) is small and well below the 5th percentile background exposure range. This risk-specific concentration is so small relative to the variability in total equivalent concentrations that it represents an indistinguishable increase in risk relative to background exposure and thus cannot be interpreted as presenting a meaningful additive above-background EO cancer risk. In contrast, the TCEQ high-range risk-specific concentration value is above the total equivalent background exposure concentration range at a level where one might expect to see a significant increased risk. This illustration reinforces the lack of utility USEPA risk-specific concentrations have in managing industrial EO exposures and communicating risk for near-facility populations and indicates that the TCEQ risk-specific concentrations are better aligned with total equivalent background exposure concentrations and therefore would provide utility in managing and communicating risk.

## 4. Discussion

Previous assessments to quantify U.S. population background exposure to EO have shown that background exposure is large relative to risk-specific concentrations from USEPA’s 2016 reassessment of cancer potency, thus limiting the utility of assessment-inferred risk-specific concentrations to effectively manage and communicate excess risk for populations residing near emitting industrial facilities [1,9,10,11]. Several sources of new data have become available since the previous background assessments were published. This updated assessment was conducted to refine the characterization of U.S. general population background exposures to EO and apply this background exposure information as a means to better understand the health significance context of the added ambient EO exposures potentially experienced by populations residing near industrial facilities.

This update includes the latest data from NHANES for HEV biomarkers that reflect total exposure to EO, as well as the latest post-refinement monitoring data for EO in ambient air away from emitting industrial facilities based on refined USEPA sampling methods and with biased samples removed. The results of this update continue to show that exogenous exposures to EO in ambient air (Pathway 2) are relatively minor contributors to total background exposure. Some uncertainty is introduced into the assessment from the data used to quantify exogenous exposures to ET, which although they reflect the best data available are less recent (i.e., collected in 2005–2006 during the Windsor exposure study conducted by Health Canada, 2010 [19]) than the data used to characterize total exposure and exogenous exposure to EO in air. More recent monitoring data for ET in air would serve to increase confidence in quantifying the contributions of this pathway in this assessment. In nonsmokers, total exposure to EO is primarily attributed to endogenous production (Pathway 1), while total exposure to EO in smokers is primarily attributed to their smoking (Pathway 3), followed by endogenous production. High confidence is given to the background TE and EE values provided in Table 5 since they rely on high-quality datasets (CDC’s HEV data from NHANES, USEPA’s air monitoring data for EO), a verified continuously linear relationship between HEV and EO exposure from low-ppb to high-ppm exposures, and a scientifically sound conceptual model (Figure 1). Variation in the TE and EE estimates in Table 6 is consistent with the identified sources of the variation in the endogenous production of EO, in which variation in gut microflora is highly variable in humans and affected by diet (e.g., probiotics, food consumption), geographic location, medication (e.g., antibiotic use), disease states, and other factors (e.g., age, gender, race) (as discussed in Kirman et al. [1]). Additional analyses of the NHANES dataset [12] with respect to dietary factors, disease states, as well as demographic and other factors could be performed to further characterize the variation in EO exposure of specific subpopulations within the U.S. but are beyond the scope of the present work.

**Figure 8:**
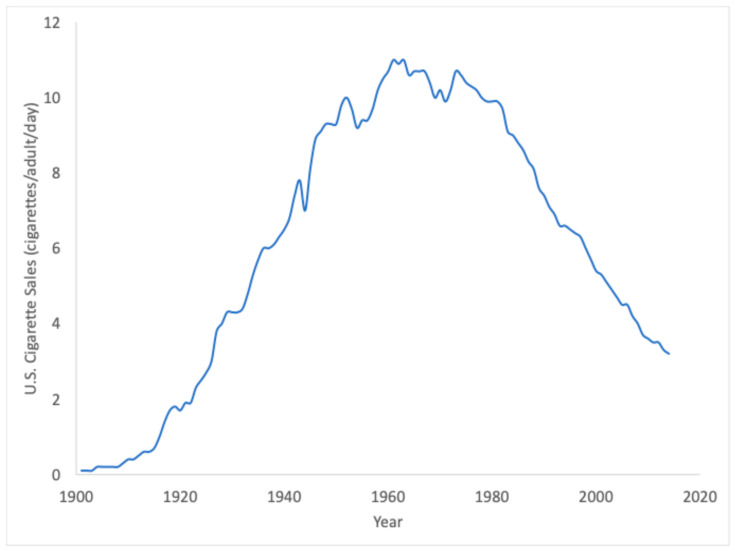
Historical cigarette sales in the U.S. reflect over 100 years of exogenous exposure to EO from smoking (Pathway 3) (https://ourworldindata.org/smoking; accessed 11 June 2024).

In this assessment, equivalent air exposures for smoking exposures (Pathway 3) were also provided, corresponding to U.S. smokers (Table 7), which on average correspond to exposures to 16.6 ppm EO in air, and range from approximately 1.2 ppb for light smokers to approximately 58.4 ppb for heavy smokers. Importantly, these smoking-related exposures were calculated from direct analytical measurements of EO emitted per cigarette smoked correlated to EO HEV adducts. These values are four orders of magnitude higher than the 10^−5^ risk-specific concentration value calculated from the USEPA cancer potency estimate for EO. Through smoking (Pathway 3), the U.S. has more than 100 years of historical exposure to EO, as indicated by historical sales data for cigarettes (Figure 8). CDC’s HEV data were collected during a period during which smoking exposures to EO have waned considerably from their peak in the 1960s. Despite this long history of elevated EO exposures to a substantial fraction of the U.S. population, the evidence supporting a causal relationship between smoking and EO-related cancers (lymphohematopoietic cancer, primarily non-Hodgkin lymphoma or NHL, myeloma, and lymphocytic leukemia; breast cancer) is not strong. In the U.S. Surgeon General’s report “*The Health Consequences of Smoking—50 Years of Progress*”, a causal relationship was considered “sufficient” for only one subcategory of lymphohematopoietic cancer (acute myeloid leukemia or AML) [50]. However, myeloid leukemia mortality was not increased in EO workers [51]. In contrast, the Surgeon General makes no conclusions on any causal associations (i.e., neither “sufficient” nor “suggestive) for smoking and the specific subcategories of lymphohematopoietic cancer (NHL, myeloma, lymphocytic leukemia) associated with exposure in EO workers. With respect to breast cancer, the Surgeon General upgraded the conclusion from “no causal relationship” [52] to “suggestive but not sufficient” to infer a causal relationship with smoking [50]. For both endpoints, the historical data for smoking appear to be inconsistent with USEPA’s large cancer potency estimate for low exposures to EO. As such, the human experience with EO exposure from smoking might serve as an additional resource for assessing and managing the potential risks from low exposures to EO, either through the identification of a no-observed-adverse-effect-level (NOAEL) to serve as an alternative point of departure for cancer risk and/or to serve as a validation dataset that could be used to place bounds upon future estimates of cancer potency for EO.

One of the primary criticisms of previous TE and EE assessments was the reliance upon an assumed linear relationship between EO exposure and HEV biomarker burden. Multiple lines of evidence were assessed herein to support the validity of this relationship. First, a linear regression analysis of HEV measurements in workers with known exposures to EO provides empirical support at moderate to high exposure levels (Figure 3). Theoretical support for a linear relationship is provided by the predictions of different models for EO (toxicokinetic model of DFG, [22]; PBPK model of Filser and Klein, [20]). Lastly, and importantly, the linear regression of HEV measurements in U.S. smokers supports a conclusion that the linear relationship between HEV and EO exposures (via tobacco smoke) continues downward from moderate exposure levels in U.S. smokers to low exposure levels in U.S. nonsmokers. As such, the linear relationship between EO total background exposure and HEV biomarker burdens is strongly supported and should be considered factually substantiated by the weight of evidence. This relationship is expected to continue within the range of background EO exposures. As a useful biomarker of exposure, it is reasonable to infer that individuals corresponding to the 1st and 99th percentile values for HEV in U.S. nonsmokers (5.8 vs. 76.9 pmol/g hemoglobin) experience internal doses of EO that differ by a similar magnitude (i.e., approximately 13-fold). USEPA’s skepticism on the linear toxicokinetics of EO [5] is unusual and ironic given that low-dose linearity serves as the default assumption for toxicokinetics and also serves as the default assumption for toxicokinetics and toxicodynamic factors for the low-dose risk of carcinogens [53].

In addition, the following criticisms from USEPA [5] of past EE calculations do not detract from the high confidence in the present assessment.

- Lack of Direct Endogenous EO Measurements—Direct measurements of endogenous EO levels are not required to quantify this pathway. Direct measurements are available for endogenous ET from closed-chamber studies of human volunteers [54,55], whose data were considered in the PBPK model developed for EO [20] and for which EO production from ET metabolism is consistent with toxicokinetic principles and model predictions (Figure 3). Hemoglobin biomarkers such as HEV serve as much better biomarkers of EO exposure than direct blood EO measurements, since they are cumulative biomarkers that reflect integrated exposures over several months prior to measurement, making them the biomarker of choice of EO for health agencies (e.g., CDC, DFG). More importantly, direct measurements for exogenous EO (USEPA’s air monitoring data) and for exogenous ET (Health Canada air monitoring data) account for only a fraction of the direct measurements made for total exposures to EO (CDC’s HEV data). Therefore, the indirect estimates of endogenous production based upon the difference between these two direct measurements of air monitoring and CDC’s total exposures remain scientifically sound.
- Other Biomarker Data Do Not Suggest a Large Endogenous Exposure—Data are also available for other biomarkers of exposure to ET, including measurements for ET in exhaled breath. USEPA [5] cites the study of Paardekooper et al. [56] as providing a baseline exhalation of ET (0.5 ppb; collected from measurements for a single breath) that does not support a large source of endogenous exposure. However, this exhalation value is inconsistent with other reported values in the published literature. For example, Popa et al. [57] reported exhalation levels of 27–29 ppb in healthy control subjects, and they historically range from 3–100 ppb in healthy individuals. Similarly, Bratu [58] reported baseline levels of ET for nose and mouth breathing of approximately 29–32 ppb. In closed-chamber studies of unexposed human volunteers, chamber concentrations of ET reached levels of approximately 20–50 ppb over a 1.5–2.5 h period [54,55]. Collectively, these data indicate that the unusually low levels reported in a single study [56] do not provide a strong argument to counter the indirect estimates for endogenous exposure obtained here using CDC’s HEV biomarker data.
- Background and Endogenous EO Exposures Are Not Integral to Estimate Extra Risk—the USEPA [5] asserts that endogenous and total background exposures to EO are not integral to extra risk determinations and therefore have no relevance to regulatory decision making. While true that both USEPA’s and TCEQ’s cancer potency derivation for EO does not need to include the consideration of these pathways, this is not the case when it comes to risk management and risk communication. By analogy to characterizations of background metals in soil, background comparisons are typically made for exposures to naturally occurring metals such as arsenic, chromium, and beryllium that may be present in soil [59], despite the fact that the cancer potency estimates for these metals are also derived in terms of extra risk [60]. The TE and EE values in Table 6 are intended to provide EO exposure comparisons that are analogous to USEPA’s approach for soils [59], in providing several options for identifying what level constitutes a “substantial difference” from the background, including (1) multiples of the mean background level, (2) multiples of the standard deviation for background levels, and (3) percentiles for the distribution of background levels.

By including Monte Carlo methods in this assessment, efforts were made to include sources of uncertainty and variation and their impact on the resulting TE and EE values. The parameter CF2 (linear relationship between HEV and EO exposure; Figure 3) was the most sensitive parameter contributing to variation in the resulting EE values. Additional work to refine this parameter, such as combining the worker HEV data (Figure 3) and the CDC HEV data (Figure 5) into a single regression analysis, could serve to reduce the variation in the TE and EE estimates. In addition, while high confidence is given to the data used to characterize the total exposure to EO (CDC’s HEV data) and exogenous EO exposure (USEPA’s air monitoring data), the data used to characterize exogenous exposures to ET are slightly older (2005–2006) and from the U.S./Canadian border, and therefore the assessment would benefit from more recent air monitoring data for ET within the U.S.

USEPA’s 1 × 10^−4^ risk-specific concentration was shown to be well below the U.S. nonsmoking population 5th to 95th percentile background TE exposure concentration range. This risk-specific concentration (0.011 ppb) is so small that it cannot be distinguished from the variability in population background concentrations, and thus the USEPA risk-specific concentrations lack utility in managing and communicating EO risk from industrially exposed populations. In contrast, the TCEQ 1 × 10^−4^ risk-specific concentration (24 ppb) is outside the nonsmoking population TE and EE background concentration range, thus indicating that the TCEQ risk-specific concentrations could provide risk management utility for highly exposed near-facility populations. In the absence of a useful USEPA risk-specific concentration for risk management purposes, the U.S. nonsmoking population background TE exposure concentration distribution as an alternative can provide context to the health significance of local ambient EO concentration above-background levels for potentially exposed near-facility populations.

## 5. Conclusions

The updated background TE and EE concentration distributions were derived using verified methods and quality data. These concentrations appear to be representative of the levels U.S. populations have experienced from the beginning of this century (the historic date range of EO exposure data incorporated into the TE and EE concentration derivation). The TE and EE estimates of U.S. nonsmoking population background exposure concentrations provide context for interpreting the significance of potential exogenous exposures greater than the ambient background and provide value as a reality check on the utility of agency cancer risk assessment risk-specific concentrations in managing and communicating the risk of excess exogenous EO exposures potentially experienced by populations residing near emitting industrial facilities. Based on the range of nonsmoking background exposures, only the TCEQ risk-specific concentrations, at present, provide utility in managing exposures for populations residing near industrial facilities emitting EO.

## Figures and Tables

**Table 1:** Total HEV in U.S. smokers and nonsmokers 2013–2020 [12].

		HEV Concentration (pmol/g Hemoglobin) **			
Population *	Detection Frequency	Detected Range	Arithmetic Mean	Standard Deviation	Percentiles
Nonsmokers (serum cotinine ≤10 μg/L)	6415/6695 (95.8%)	8.27–656	22.9	17.8	P01 = 5.8 P05 = 9.2 P10 = 12.1 P25 = 15.4 P40 = 17.9 P50 = 19.7 P60 = 21.7 P75 = 25.9 P90 = 34.8 P95 = 42.2 P99 = 76.9
Smokers (serum cotinine > 10 μg/L)	1423/1428 (99.6%)	8.59–1460	187	150	P01 = 13.4 P05 = 22.3 P10 = 36.4 P25 = 83.3 P40 = 127.1 P50 = 154.4 P60 = 186.9 P75 = 249.7 P90 = 370.5 P95 = 459.2 P99 = 660.0

* Smoking status defined using serum cotinine: nonsmokers <= 10 ng/mL; smokers > 10 ng/mL; ** statistics were calculated using the detection limit divided by 2 for all non-detect samples.

**Table 2:** Description of raw ambient EO monitoring data from the USEPA AQS database [18] for the period 2019 to 2023, by year and pre-refinement (2019–2020) and post-refinement (2021–2023) periods.

	Pre-Refinement ^2^		Post-Refinement ^3^		
Descriptor	2019	2020	2021	2022	2023
Unique States Reporting Data (N)	11	19	20	19	22
Unique Site Locations Reporting Data (N)	26	45	61	56	56
Collection Method: Passive (N) [% non-detect] ^1^	696 [11.8]	1173 [6.4]	1967 [11.3]	2089 [18.9]	1762 [11.0]
Collection Method: Pressurized (N) [% non-detect] ^1^	355 [8.5]	989 [10.9]	1470 [13.9]	1200 [11.2]	1117 [3.0]

^1^ Defined as concentrations reported as 0 ppb; ^2^ monitoring before method refinements were introduced (pre-refinement); ^3^ monitoring after method refinements were included in sampling (post-refinement).

**Table 3:** Parameter distributions used for calculating endogenous HEV burden (HEVE) and equivalent air concentrations.

Parameter (Abbreviation)	Units	Distribution	Basis
Total hydroxyethylvaline (HEVTns)	pmol/g hemoglobin	Custom Distribution based on percentiles: Min = 5.8 Max = 280 * P01 = 5.8 P05 = 9.2 P10 = 12.1 P25 = 15.4 P40 = 17.9 P50 = 19.7 P60 = 21.7 P75 = 25.9 P90 = 34.8 P95 = 42.2 P99 = 76.9	Distribution is based on HEV measurements made for U.S. nonsmokers as reported by CDC NHANES (for 2013–2020 combined). Distribution reflects temporal and interindividual variation across various demographic, genetic, and health factors.
Concentration of EO in air (EOair)	ppb (continuous exposure)	Custom distribution based on percentiles: Min = 0.015 Max = 0.65 P01 = 0.015 P05 = 0.015 P10 = 0.015 P25 = 0.030 P50 = 0.050 P75 = 0.080 P90 = 0.11 P95 = 0.15 P99 = 0.40	Distribution is based on ambient air concentrations of EO from USEPA air monitoring data. Distribution reflects daily temporal and geospatial variation and was used to characterize variation in the mean concentration experienced over the lifetime of an erythrocyte (120 days). Based on this simulation, the 120-day average exposure was characterized as a normal distribution (mean = 0.068, SD = 0.0066).
Concentration of ethylene in air (ETair)	ppb (continuous exposure)	Custom distribution based on percentiles: Min = 0.94 Max = 105 P05 = 1.4 P25 = 2.3 P50 = 3.1 P75 = 4.7 P90 = 9.0 P95 = 13.6	Distribution is based on personal air concentrations of ET from Health Canada air monitoring data [19]. Distribution reflects daily temporal and interindividual variation, and was used to characterize variation in the mean concentration experienced over the lifetime of an erythrocyte (120 days). Based on this simulation the 120-day average exposure was characterized as a normal distribution (mean = 6.6, SD = 1.3).
Conversion Factor 1: Metabolic conversion fraction of ET to EO (CF1)	Unitless	Pert (0.005, 0.0125, 0.05)	Best estimate reflects predictions by the PBPK model of Filser and Klein [20] for humans exposed to either EO or ET; the upper limit is based upon the published estimate of Csanady et al. (2000) [21]; the lower limit is based on professional judgement. Distribution reflects uncertainty in the fraction of ET that is metabolically converted to EO within the body.
Conversion Factor 2: Correlation slope for HEV as a function of EO exposure (CF2)	pmol/g hemoglobin per ppb continuous exposure	Normal (11.3, 4.2)	Range reflects the uncertainty in the slope term based upon a robust errors linear regression of worker HEV data (Figure 3). The slope was converted to a continuous exposure basis adjusted for worker exposure frequency (250 vs. 365 days/year) and breathing rate (10 vs. 20 m^3^/day). Distribution encompasses slopes from pharmacokinetic model predictions (DFG 1994) [22] and PBPK modeling predictions [17].

* Excludes two samples identified as outliers.

**Table 4:** Summary of the publicly available data from the measurement of ambient EO concentrations in Georgia and Utah in the vicinity of sterilization facilities and at background locations.

Facility	Location	Monitoring Dates	*n* (Sites) ^1^	Dist. ^2^
Sterigenics [39]	Smyrna, GA	1/2021–9/2022	141 (6)	~260–1640
Becton Dickinson [40]	Covington, GA	1/2021–9/2022	139 (5)	~110–2370
Sterilization Services [41]	Atlanta, GA	1/2021–9/2022	106 (3)	~110–5630
Background [42]	General Coffee, GA	1/2021–9/2022	26 (1)	>290,000
Background [43]	South DeKalb, GA	1/2021–9/2022	175 (1)	>22,500
BD Medical [44]	Salt Lake City, UT	1–3, 7–9/2022	109, 81 (8)	~180–900
Sterigenics [44]	Salt Lake City, UT	1–3, 7/9/2022	47, 28 (4)	~230–700
Background [44]	Salt Lake City, UT	1–3, 7–9/2022	53, 62 (5)	>4425

^1^ Total number of samples (total number of sampling sites); ^2^ estimated distance of sample sites from the facility in meters.

**Table 5:** Summary of clean ambient background EO concentrations from monitoring for the period 2019 to 2023, by year, combined pre-refinement, combined post-refinement, and collection method.

	2019		2020		2019–2020 Pre-Refinement		2021		2022		2023		2021–2023 Post-Refinement	
Statistic	Passive	Pressurized	Passive	Pressurized	Passive	Pressurized	Passive	Pressurized	Passive	Pressurized	Passive	Pressurized	Passive	Pressurized
N	347	290	698	929	1045	1219	1318	1315	1552	1046	1222	979	4092	3340
Mean (St. Dev.)	0.113 (0.099)	0.080 (0.054)	0.076 (0.085)	0.075 (0.058)	0.088 (0.092)	0.076 (0.057)	0.064 (0.068)	0.069 (0.063)	0.068 (0.070)	0.070 (0.062)	0.064 (0.062)	0.079 (0.072)	0.066 (0.067)	0.072 (0.065)
5th Percentile	0.015	0.015	0.015	0.015	0.015	0.015	0.015	0.015	0.015	0.015	0.015	0.020	0.015	0.015
10th Percentile	0.015	0.015	0.015	0.015	0.015	0.015	0.015	0.015	0.015	0.015	0.015	0.030	0.015	0.015
25th Percentile	0.03	0.050	0.050	0.045	0.050	0.045	0.035	0.040	0.015	0.040	0.040	0.040	0.030	0.040
50th Percentile (Median)	0.085	0.070	0.050	0.065	0.050	0.065	0.050	0.055	0.050	0.060	0.050	0.060	0.050	0.060
75th Percentile	0.158	0.100	0.055	0.090	0.100	0.093	0.070	0.080	0.090	0.085	0.080	0.090	0.080	0.085
90th Percentile	0.245	0.130	0.120	0.125	0.200	0.130	0.100	0.123	0.130	0.115	0.100	0.120	0.110	0.120
95th Percentile	0.320	0.165	0.250	0.165	0.283	0.165	0.150	0.170	0.157	0.139	0.140	0.190	0.150	0.165

**Table 6:** Equivalent exposure concentrations of EO in air corresponding to total and endogenous hemoglobin adduct burdens in nonsmokers.

		Hemoglobin Adduct (pmol/g)		Equivalent Exogenous EO Exposure (ppb, Continuous)	
Basis	Multiplier or Percentile	HEVTns	HEVE, Attributed to Endogenous Pathways (90% CI)	TE, Corresponding to Total HEV (90% CI)	EE, Corresponding to Endogenous HEV (90% CI)
Multiples of HEVTns Mean (22.9 pmol/g)	0.1	2.3	2.1 (1.9–2.2)	0.35 (0.13–0.52)	0.33 (0.11–0.5)
	0.25	5.7	5.2 (4.7–5.6)	0.87 (0.31–1.3)	0.82 (0.27–1.3)
	0.5	11.5	10.4 (9.4–11.1)	1.7 (0.63–2.6)	1.6 (0.53–2.5)
	1	22.9	20.8 (18.9–22.2)	3.5 (1.3–5.2)	3.3 (1.1–5)
	2	45.8	41.6 (37.8–44.4)	7 (2.5–10.4)	6.6 (2.1–10.1)
	3	68.7	62.4 (56.6–66.6)	10.4 (3.8–15.6)	9.9 (3.2–15.1)
Multiples of HEVTns SD (17.8 pmol/g)	0.1	1.8	1.6 (1.4–1.7)	0.3 (0.1–0.4)	0.3 (0.1–0.4)
	0.25	4.5	3.9 (3.4–4.3)	0.7 (0.2–1)	0.6 (0.2–1)
	0.5	8.9	7.9 (6.9–8.6)	1.4 (0.5–2)	1.3 (0.4–1.9)
	1	17.8	15.7 (13.8–17.1)	2.7 (1–4.1)	2.5 (0.8–3.9)
	2	35.6	31.4 (27.6–34.2)	5.4 (2–8.1)	5 (1.6–7.7)
	3	53.4	47.1 (41.3–51.3)	8.1 (2.9–12.2)	7.6 (2.4–11.6)
Percentiles for HEVTns	0.01	5.8	3.7 (1.8–5.1)	0.9 (0.3–1.3)	0.7 (0.1–1.1)
	0.05	9.2	7.1 (5.2–8.5)	1.4 (0.5–2.1)	1.2 (0.3–1.9)
	0.1	12.1	10 (8.1–11.4)	1.8 (0.7–2.8)	1.7 (0.5–2.6)
	0.25	15.4	13.3 (11.4–14.7)	2.3 (0.8–3.5)	2.2 (0.7–3.3)
	0.4	17.9	15.8 (13.9–17.2)	2.7 (1–4.1)	2.5 (0.8–3.9)
	0.5	19.7	17.6 (15.7–19)	3 (1.1–4.5)	2.8 (0.9–4.3)
	0.6	21.7	19.6 (17.7–21)	3.3 (1.2–4.9)	3.1 (1–4.8)
	0.75	25.9	23.8 (21.8–25.2)	3.9 (1.4–5.9)	3.7 (1.2–5.7)
	0.9	34.8	32.7 (30.8–34.1)	5.3 (1.9–7.9)	5.1 (1.7–7.7)
	0.95	42.2	40.1 (38.2–41.5)	6.4 (2.3–9.6)	6.2 (2.1–9.4)
	0.99	76.9	74.8 (72.9–76.2)	11.7 (4.2–17.5)	11.5 (4–17.3)

**Table 7:** Total equivalent concentrations of EO in air corresponding to U.S. smoker total HEV burdens.

Statistic	Total HEV (pmol/g) (CDC, 2024)	Total Equivalent Exposure to EO (ppb, Continuous)
mean	187.1	16.6
P01	13.4	1.2
P05	22.3	2.0
P10	36.4	3.2
P25	83.3	7.4
P40	127.1	11.2
P50	154.4	13.7
P60	186.9	16.5
P75	249.7	22.1
P90	370.5	32.8
P95	459.2	40.6
P99	660.0	58.4

**Table 8:** Measures of central tendency and spread for ambient air EO concentrations in monitoring locations in the vicinity of three sterilization facilities in Georgia are summarized, and significant statistical comparisons of near-facility and local and national background locations are identified.

Facility	Sample Site (*n*) ^1^	Distance ^2^	2021 Mean (SD) ^3,4,5,6^	2021 P50	2022 Mean (SD) ^3,4,5,6^	2022 P50
			ppb	ppb	ppb	ppb
Georgia						
Sterigenics	S1 (22)	~1420 sw	0.109 (0.054)	0.097		
	S2 (52)	~1640 w	0.098 (0.053) ^6^	0.084		
	S3 (21)	~910 e	0.175 (0.132) ^4,5^	0.141		
	S4 (18)	~260 n	0.120 (0.067)	0.103		
	S6 (12)	~1570 nw	0.136 (0.042) ^4^	0.130	0.160 (0.257)	0.093
	S7 (16)	~1350 nw	0.254 (0.230) ^6^	0.182	0.083 (0.030)	0.076
Becton Dickinson	C2 (56)	~1020 se	0.133 (0.100) ^4,6^	0.108	0.089 (0.043) ^4,6^	0.108
	C3 (23)	~730 s	0.175 (0.239)	0.113		
	C4 (22)	~800 w	0.138 (0.084)	0.115		
	C5 (22)	~330 s	0.154 (0.172)	0.104		
	C7 (7)	~400 se	0.224 (0.242) ^6^	0.150		
	C7SA/C3SA (9)	~630 s			0.094 (0.055)	0.082
Sterilization Services	F1 (34)	~830 se	0.152 (0.089) ^4,6^	0.128	0.096 (0.059)	0.079
	F2 (55)	~340 se	0.213 (0.164) ^4,5,6^	0.173	0.171 (0.221) ^4,5,6^	0.121
	F4 (17)	~5360 nw	0.109 (0.086)	0.089		
Background	GC (26)	>290,000	0.056 (0.025)	0.051	0.049 (0.021)	0.046
	SD (1750)	>22,500	0.098 (0.058)	0.085	0.073 (0.027)	0.068
	USPA		0.064 (0.068)		0.069 (0.063)	
	USPR		0.068 (0.070)		0.070 (0.062)	

^1^ Sample sites labeled by Georgia EPD or Utah Division of Air Quality and total number of samples collected at that sample site; ^2^ estimated distance of sample sites from facility in meters and direction from the emission source; ^3^ SD, standard deviation; ^4^ significantly different from background (General Coffee Park) when data were first log-transformed and then compared in *t*-tests, incorporating a Bonferroni correction (alpha = 0.0024) to adjust for multiple comparisons; ^5^ significantly different from the South DeKalb background when data were first log-transformed and then compared in *t*-tests, incorporating a Bonferroni correction (alpha = 0.0024) to adjust for multiple comparisons; ^6^ significantly different from the U.S. background when data were first log-transformed and then compared in *t*-tests, incorporating a Bonferroni correction (alpha = 0.0024) to adjust for multiple comparisons. Similar results were observed when comparing medians in the Wilcoxon rank-sum tests, again with a Bonferroni correction.

**Table 9:** Measures of central tendency and spread for ambient air EO concentrations in monitoring locations in the vicinity of two sterilization facilities in Utah are summarized, and significant statistical comparisons of near-facility and local background locations are identified.

Facility	Sample Site (*n*) ^1^	Distance ^2^	Summer Mean (SD) ^3,4^	P50	Winter Mean (SD) ^3,4^	P50
			ppb	ppb	ppb	ppb
Utah						
Sterigenics	SG1 (13, 8)	~285 se	0.236 (0.099) ^4^	0.214	0.121 (0.063) ^4^	0.106
	SG2 (12, 7)	~450 nw	0.419 (0.025) ^4^	0.340	0.091 (0.053)	0.077
	SG3 (12, 13)	~700 n	0.173 (0.074)	0.157	0.067 (0.031)	0.061
	SG4 (10, -)	~230 sw	0.340 (0.284) ^4^	0.272		
BD Medical	BD1 (13, 6)	~180 w	0.405 (0.301) ^4^	0.321	0.127 (0.063) ^4^	0.113
	BD2 (12, 12)	~300 w	0.258 (0.123) ^4^	0.230	0.074 (0.043)	0.066
	BD3 (13, 7)	~300 s	0.344 (0.306) ^4^	0.263	0.088 (0.055)	0.078
	BD4 (16, 13)	~780 s	0.129 (0.043)	0.123	0.050 (0.013)	0048
	BD5 (14, 13)	~900 s	0.192 (0.076)	0.177	0.058 (0.012)	0.057
	BD6 (13, 9)	~500 se	0.496 (1.25)	0.183	0.069 (0.029)	0.064
	BD7 (14, 9)	~690 ne	0.203 (0.174)	0.169	0.048 (0.012)	0.047
	BD8 (14, 12)	~1400 nw	0.169 (0.121)	0.143	0.047 (0.013)	0.046
Background	BG1 (14, 11)	>1850	0.130 (0.048)	0.123	0.044 (0.019)	0.041
	BG2 (12, 13)	>1850	0.107 (0.058)	0.095	0.047 (0.017)	0.044
	BG3 (12, 12)	>1850	0.142 (0.055)	0.133	0.053 (0.012)	0.052
	BG4 (15, 13)	>1850	0.135 (0.057)	0.125	0.059 (0.009)	0.058
	BG5 (-, 13)	>1850			0.045 (0.018)	0.043
	BG1-5 S (53)	>1850	0.129 (0.054)	0.119		
	BG1-5 W (62)	>1850			0.050 (0.016)	0.047

^1^ Sample sites labeled by the Utah Division of Air Quality and total number of samples collected at that sample site (summer, winter); ^2^ estimated distance of sample sites from the facility in meters and the direction from emission source; ^3^ SD, standard deviation; ^4^ significantly different from background when data were first log-transformed and then compared in *t*-tests, incorporating a Bonferroni correction (alpha = 0.0022) to adjust for multiple comparisons. Similar results were observed when comparing medians in the Wilcoxon rank-sum tests, again with a Bonferroni correction.

## Data Availability

Publicly available datasets were analyzed in this study. These data can be found in the References section.
